# Local Deformation of Glasses is Mediated by Rigidity Fluctuation on Nanometer Scale

**DOI:** 10.1002/advs.201800916

**Published:** 2018-08-29

**Authors:** Omar Benzine, Sebastian Bruns, Zhiwen Pan, Karsten Durst, Lothar Wondraczek

**Affiliations:** ^1^ Otto Schott Institute of Materials Research University of Jena Fraunhoferstrasse 6 07743 Jena Germany; ^2^ Department of Materials Science Physical Metallurgy Technical University of Darmstadt Alarich‐Weiss‐Straße 2 64287 Darmstadt Germany; ^3^ Abbe Center of Photonics University of Jena Albert‐Einstein‐Strasse 6 07745 Jena Germany

**Keywords:** deformations, flexible substrates, glass, heterogeneity, silica, strength

## Abstract

Microscopic deformation processes determine defect formation on glass surfaces and, thus, the material's resistance to mechanical failure. While the macroscopic strength of most glasses is not directly dependent on material composition, local deformation and flaw initiation are strongly affected by chemistry and atomic arrangement. Aside from empirical insight, however, the structural origin of the fundamental deformation modes remains largely unknown. Experimental methods that probe parameters on short or intermediate length‐scale such as atom–atom or superstructural correlations are typically applied in the absence of alternatives. Drawing on recent experimental advances, spatially resolved Raman spectroscopy is now used in the THz‐gap for mapping local changes in the low‐frequency vibrational density of states. From direct observation of deformation‐induced variations on the characteristic length‐scale of molecular heterogeneity, it is revealed that rigidity fluctuation mediates the deformation process of inorganic glasses. Molecular field approximations, which are based solely on the observation of short‐range (interatomic) interactions, fail in the prediction of mechanical behavior. Instead, glasses appear to respond to local mechanical contact in a way that is similar to that of granular media with high intergranular cohesion.

## 1 Introduction

The mechanical behavior of glasses and their surfaces has become a subject of widespread interest.^1^ Most prominently, this has been triggered by rapidly emerging products which rely on haptic interaction and, hence, are prone to surface damage, for example, touch panels, personal electronic devices, and thin and flexible displays. Overcoming the simplistic notion of ultimate brittleness, it has now been understood that the early‐stage resistance to defects can be tailored through chemical composition, and glasses with improved damage resistance have been identified. However, understanding plastic deformation in glasses has been a challenging task. Due to the absence of structural periodicity, simple concepts of dislocation movement, or shear band formation cannot readily be applied. This lack of mechanistic knowledge still limits conceptual tools for material design.

Since the first report on plastic deformation of optical glasses,^2^ the seminal subsequent differentiation into “normal” and “anomalous” glasses^3^ and early intentional developments of silicate glasses with reduced brittleness,^4^ a large number of studies has been relying on micro‐ and nanoindentation to elucidate the interplay between glass chemistry and the material's response in sharp‐contact situations.^5^, ^6^ Focusing on vitreous silica as an archetypal model, Perriot et al.^7^ were the first to carry‐out Raman spectroscopic mapping of Vickers indentation residual imprints. Drawing on studies of Suguira et al.^8^ which revealed an analytical relationship between the shift of the characteristic D2 Raman band and the degree of structural densification, they found a heterogeneous distribution of densification around the residual imprint. Similar work was subsequently conducted on other commercially relevant glasses,^9^, ^10^ and extended by Tran et al.^11^ through 3D micro‐Brillouin mapping.

Such experimental mapping data have led to the development of constitutive descriptions of the indentation process^12^, ^13^, ^14^, ^15^ and associated cracking phenomena.^16^ However, a view at the underlying structural reactions was obtained only on the short‐range level, for example, demonstrating variations in the first coordination shell of network‐forming ion species. Sometimes, such observations allow for the formulation of a shear mechanism on molecular scale.^17^, ^18^ In the absence of further insight, short‐range parameters have therefore commonly been taken as indicators for the structural origin of mechanical behavior.^1^, ^19^ On the other hand, it has been shown that short‐range structural parameters are often insufficient to explain the quantitative extent of macroscopic densification and shear.^20^, ^21^

In order to bridge the gap between structural observations on short range and macroscopic behavior, we now assess the material's response to mechanical contact on intermediate (nanometer) length‐scale, considering deformation‐induced variations in the low‐frequency vibrational anomaly of glasses.

As an apparently universal feature, glassy materials exhibit an excess in the vibrational density of states (VDoS) as compared to the Debye model^22^ of crystalline materials. This excess manifests in the form of a broad band in the reduced vibrational density of states *g*(ω)/ω^2^, known as Boson peak and observable in the frequency regime of ≈10–100 cm^−1^ (0.1–3 THz), for example, by Raman spectroscopy^23^ or low‐temperature calorimetry.^24^ Recent studies (still focusing mostly on vitreous silica) have been extending the experimental perspective using hyper‐Raman spectroscopy,^25^, ^26^ far‐infrared spectroscopy,^27^ terahertz time‐domain spectroscopy,^28^ or inelastic scattering of X‐rays^29^ and neutrons.^30^

The origin of the Boson peak remains debated, mostly between three general aspects^31^, ^32^: spatially fluctuating elastic constants,^34^, ^35^ dynamic heterogeneity and the glass transition,^36^ and coupling of sound waves to quasi‐localized modes of defect states.^37^, ^38^ In all three considerations, the Boson peak is directly or indirectly related to structural heterogeneity of the solid glass, typically on the scale of a few nanometers. It has further been shown extensively that its frequency (ω_BP_) and intensity (*I*_BP_) depend not only on glass composition,^39^ but also on thermal history,^40^, ^41^ pressure^42^, ^43^, ^44^, ^45^, ^46^, ^47^, ^48^ and the presence of different polymorphic states.^49^, ^50^ Regarding the pressure‐dependence, analyses have been carried‐out primarily in quasi‐isostatic conditions, in situ using diamond anvil cells or ex situ through high temperature/high pressure multianvil set‐ups.^32^, ^39^

**Figure 1:**
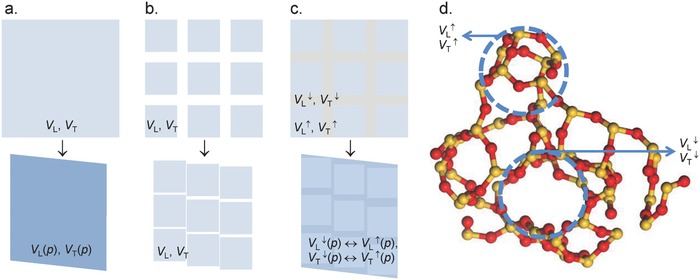
Structural heterogeneity and deformation. a–c) A continuous medium with sound velocities *V*_L_ and *V*_T_, changing to *V*_L_(*p*) and *V*_T_(*p*) upon compression and shear deformation, a granular medium in which compression consumes free volume without affecting the elastic properties of the grains, and a granular medium with an adherent intergranular region in which the rigidity contrast between grains and the intergranular region is reduced during deformation. d) The presence of structural heterogeneity and rigidity fluctuation in vitreous silica is rationalized on a MD trajectory of amorphous SiO_2_ (adapted from ref. 50) highlighting regions of high and low network density which alternate on the scale of a few nanometers (yellow balls: Si; red balls: O). The marked network regions have a diameter of ≈1 nm.

In analogy to lateral or 3D mapping of short‐range order during indentation deformation, and using isostatic compaction data for reference, we now map variations on the Boson peak as they result from local structural deformation. Using low‐frequency Raman spectroscopy for facile data acquisition in combination with a recently introduced constitutive model for glass densification, we reveal how local variations in the elastic properties and, hence, network rigidity respond to microscopic material deformation. From this, we argue that structural heterogeneity and fluctuations in molecular rigidity drive the deformation process. That is, continuous medium and molecular field approximations (**Figure** **1**a) which are based alone on the observation of short‐range (interatomic) interactions fail in the prediction of mechanical behavior. Instead, glasses appear to respond to local mechanical contact in a way which is similar to that of granular media (Figure 1b), whereby grain analogues occur in the form of regions of high structural rigidity which are embedded in a less rigid matrix (Figure 1c). The origin of such fluctuations in network rigidity is fluctuations in bond energy density, manifest in the presence of structural voids, and denser agglomerates of superstructural units (Figure 1d).

## 2 Results

### 2.1 Raman Scattering and Local Densification

**Figure 2:**
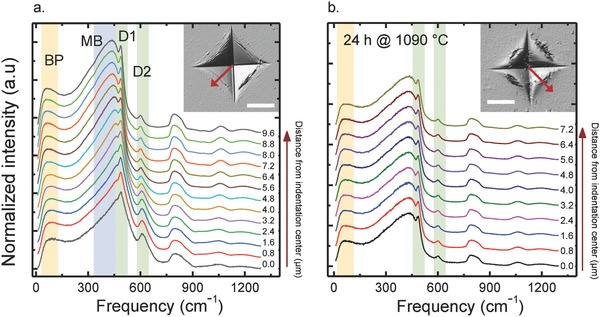
Raman scattering of vitreous silica after indentation deformation. Raman spectra were collected by scanning along the top‐view of a Vickers indent (created by loading with 2.94 N for 15 s) as indicated in the insets, before a) and after annealing at ≈0.9 *T*_g_ for 24 h. Curves show the Stokes‐side of normalized Raman spectra recorded at a step‐width of 0.4 µm. For better visibility, we display only every second curve (0.8 µm spacing). The locations of the Boson peak (BP), the silica main band (MB), and the defect bands D1 and D2 are highlighted. Scale bars in the insets are 10 µm.

Normalized Raman scattering spectra as obtained from the top face of a silica glass sample after Vickers indentation are shown in **Figure** **2**a. All spectra exhibit the characteristic band shape of vitreous silica. It comprises of three regions: 1) the high‐frequency regime between 850 and 1400 cm^−1^, dominated by stretching vibrations of SiO_2_ subunits at ≈800, 1080, and 1200 cm^−1^,^51^ (2) the region located within 200 to about 850 cm^−1^ with the main band (MB) at ≈435 cm^−1^ (symmetric rocking of bridging oxygen species)^52^ and the two defect bands D1 and D2 at ≈490 and 601 cm^−1^ (symmetric breathing modes of four‐ and three‐membered SiO_4_ rings, respectively),^53^ and (3) the low‐frequency region of 10–200 cm^−1^ with the pronounced Boson peak (BP) located at 50–80 cm^−1^.

The Boson peak represents the collective motion of a great number of atoms.^54^ Indentation deformation of glass surfaces is generally taken as a result of two processes: densification on molecular or intermediate length‐scale, and shear flow on longer scale.^55^ The atomic packing density and Poisson's ratio indicate which of the two reactions is dominating.^55^ Here, vitreous silica with its low Poisson ratio belongs to the so‐called anomalous glasses in which a very high degree of densification is possible, i.e., up to a fraction of 85–92% of the total amount of indentation deformation.^55^ In order to judge the individual contributions of densification and shear to the deformation‐induced variation of Boson Peak frequency, low‐frequency Raman mappings are compared before (Figure 2a) and after annealing (Figure 2b, annealed for 24 h at 0.9 × *T*_g_). This assumes that structural compaction fully relaxes during annealing, whereas shear deformation does not.

While the principal Raman signature remains unchanged before and after annealing, during heat treatment, stress relaxation is accompanied by structural relaxation^56^ and the indentation‐induced structural change recovers to a large extent. This confirms previous observations that indentation deformation of silica glass occurs primarily through structural compaction.

In order to correct first‐order Raman scattering data for the influences of temperature and frequency, the measured intensity *I*_mes_(ω,*T*) is rewritten in reduced form as the product of the reduced density of states *g*(ω)/ω^2^ and the coupling coefficient *C*(ω)^57^

(1) $$I^{\mathrm{red}}\;\left(\omega \right)\;\;=\;\;\frac{I_{\mathrm{mes}}\left(\omega ,T\right)}{\left[n\left(\omega ,T\right)+1\right]\omega }\;\;\;=\;\;\;C\left(\omega \right)\frac{g\left(\omega \right)}{\omega^{2}}$$

In Equation (1), *n*(ω,*T*) = [exp(*ħω*/*kT*)^−1^]^−1^ is the Bose–Einstein population factor for frequency ω and temperature *T*, *ħ*, and *k* are the reduced Planck and Boltzmann constants, respectively. While for pristine vitreous silica, a nearly linear frequency‐dependence is usually observed for *C*(ω) over the frequency range of 0.5ω_BP_ to 1.5ω_BP_,^32^, ^58^, ^59^ Carini et al.^32^ suggested a dependency of the form *C*(ω) ∝ *ω^α^* (within 10–120 cm^−1^, avoiding quasi‐elastic scattering at <10 cm^−1^ and optical modes at >120 cm^−1^) for densified silica. The exponent α was found to vary with the degree of densification (ρ/ρ_0_) and was fit as α  =  *A* + [*B*  ×  (ρ/ρ_0_)^*C*^)] with constants *A*, *B*, and *C*. In order to derive *g*(ω)/ω^2^ from Equation (1), we now first estimate the degree of densification corresponding to each Raman spectrum. For this, we initially use an empirical relation which relates the D2 band position to the densification ratio^7^, ^8^

(2) $$0.143\log\;\left(\frac{\rho }{\rho_{0}}\right)\;\;=\;\;\;\log\left(\frac{\omega }{\omega_{0}}\right)$$

**Figure 3:**
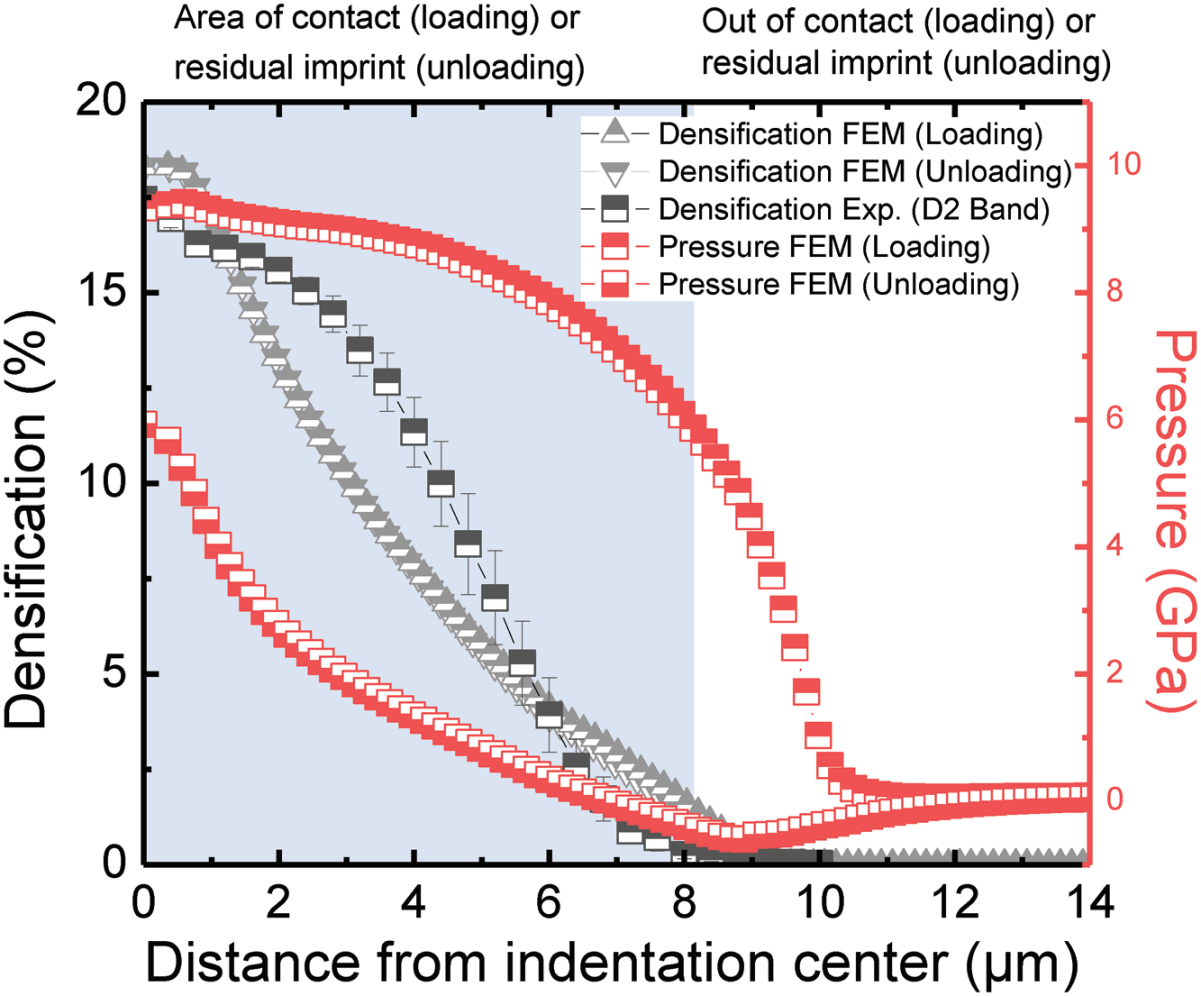
Indentation‐induced densification. Densification (PEQC4) and pressure distribution as functions of the distance from the indentation center. Experimental data are according to Equation (2). FEM simulation results are provided during holding the peak load and after unloading for a Vickers‐equivalent conical indenter with an opening angle of 70.29°. The shaded area marks the contact/imprint region.

Here, ω and ω_0_ are the observed and the reference (pristine) frequency locations of the D2 band. The evaluation through Equation (2) does not differentiate between plastic and elastic contributions.^7^ However, it has been shown that the D2 band frequency is only marginally affected by residual elastic strain.^60^ In **Figure** **3**, the thus‐obtained densification data are compared to finite element modelling (FEM) modeling results.

Finite element modeling of the indentation process reveals an inhomogeneous stress distribution beneath the indenter, both during and after loading. The equivalent pressure is largest at the indenter tip and decreases toward the edges. In the vicinity of the contact region a tensile stress component arises in radial direction, counteracting the compressive stress distribution beneath the indenter tip. This stress component causes a minimum in the equivalent pressure at a distance of roughly 9 µm. Densification is at its maximum of ≈18.3% (FEM) or 17.4% (Raman D2 experiment) right in the center of the indent. Also the lateral expansion of the compaction field matches the Raman measurements very well. In the intermediate section, densification is slightly underestimated by FEM, which might be due to the many simplifications in the model. For example, transient variations in the elastic properties are still neglected. We further notice that the maximum densification observed here is somewhat smaller than the expected saturation value of about 21% which has been reported for vitreous silica.^32^ This is similar to other observations^7^, ^12^ and may be attributed to shear flow yielding which limits the maximum hydrostatic component beneath the indenter.

### 2.2 Vibrational Density of States and Boson Peak

**Figure 4:**
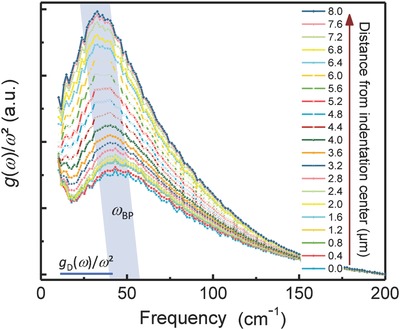
Reduced vibrational density of states *g*(ω)/ω^2^ of silica after indentation deformation. Data collected by low‐frequency Raman spectroscopy at varying distances from the center of the residual imprint (see also Figure 1). The Debye level *g*_D_/ω2 of the center spectrum is shown for reference.

From densification data, the exponent α and subsequently *C*(ω) were derived for calculating the reduced vibrational density of states, *g*(ω)/ω^2^ (**Figure** **4**).

All spectra exhibit the excess in *g*(ω) as compared to the Debye prediction which is typical for glassy materials (with $g_{D}(\omega )\;\;=\;\;3\omega^{2}/\omega_{D}^{3}\approx \;\omega^{2}$ and the Debye frequency ω_D_). In order to estimate the Boson peak frequency ω_BP_ quantitatively the reduced intensity was fitted with a log‐normal function,^39^, ^61^ taking into account the asymmetric shape of the Boson peak. In Figure 4, ω_BP_ progressively shifts to lower frequency with increasing distance from the center of the indent, i.e., from ≈45 cm^−1^ within the region of highest densification to about 33.7 cm^−1^ in a radial distance of ≈8 µm from the center of the indent. The latter value corresponds to the value of the Boson peak frequency ω_BP_ which is typically found in pristine silica (33.5 cm^−1^,^26^, ^32^, ^59^). The shift of ω_BP_ is accompanied by a significant increase of Boson peak intensity (e‐VDOS), signifying a reduction of low‐energy vibrational modes with increasing stress. Interestingly, the change in Boson peak intensity (e‐VDOS) is significantly more pronounced than the intensity variations seen on the main vibrational bands (Figure 2). This observation suggests that the low‐energy vibrational modes (Boson peak) are affected more strongly through local deformation than the higher energy vibrational modes (representing short‐range structural arrangements).

### 2.3 Indentation‐Induced Structural Compaction

The heterogeneous stress distribution shown in Figure 3 leads to an inhomogeneous distribution of densification around the residual imprint. During high‐pressure loading of 5–10 GPa, the packing density of the SiO_2_ network irreversibly increases.^62^, ^63^, ^64^ It has been suggested that this is driven by topological reconstruction on superstructural scale, in particular, affecting the distribution of rings of corner‐sharing SiO_4_ tetrahedra with different size (the proof of the existence of those rings is based mainly on Raman scattering observations of the D1 and D2 bands, Figure 2). The intertetrahedral angle θ ranges from 120° to 180° with a most frequent state of θ ≈ 144°.^65^ This wide distribution results also in a large variability of possible ring configurations, i.e., from threefolds to tenfolds,^66^, ^67^, ^68^ with sixfolds being the most frequent. For permanently densified silica, neutron diffraction has shown that the SiO_4_ tetrahedron itself is only marginally affected within the present range of pressure.^69^ Instead, the intertetrahedral angle and, thus, the Si–Si distance decrease significantly with proceeding compaction, for example, from ≈144° to about ≈138°–139° at ≈21% densification.^63^, ^64^

Considering the present observation of a reduction of the excess of low‐energy vibrational modes (Figure 4), it was suggested from INS and hyper‐Raman experiments performed on vitreous silica that low‐frequency vibrations might correspond to the rotational motions of clusters of several tetrahedra.^26^, ^70^, ^71^ According to Hehlen et al.,^26^ however, these modes are not active in infrared and Raman spectroscopy. The low‐frequency Raman signal can be interpreted within the framework of the soft potential model (SPM). SPM is assuming that the low‐frequency dynamics of glasses are characterized by the presence of additional quasi‐localized vibrations (QLV).^47^ Raman‐active QLVs could result from to the SiO_4_ librational motions coupled to the continuum of acoustic vibrations.^72^ The main effect of densification is therefore to compact the network of tetrahedra by reducing the free volume, but without affecting the elementary structural units. The decrease in the size of the rings and the reduction in dynamic degrees of freedom impede the rotational mobility of tetrahedra which are linked within the rings. The accompanying increase of network stiffness leads to the gradual suppression of soft vibrations and to an increase of their frequency ω_BP_ as observed in Figure 4.

In a similar consideration,^31^, ^34^, ^35^ random spatial fluctuations of the transverse elastic constants induce excess vibrational states at low frequency. Then, the acting pressure reduces the amplitude of fluctuation, leading to the observed shift of the Boson peak and to a decrease of its intensity relative to the Debye level: as discussed by Flores‐Ruiz and Naumis,^73^ the frequency of the Boson peak shifts to higher frequency and, at the same time, its intensity decreases with increasing network rigidity.

### 2.4 Breakdown of Continuous Medium Transformation (CMT) Predictions and Role of Rigidity Fluctuation

**Figure 5:**
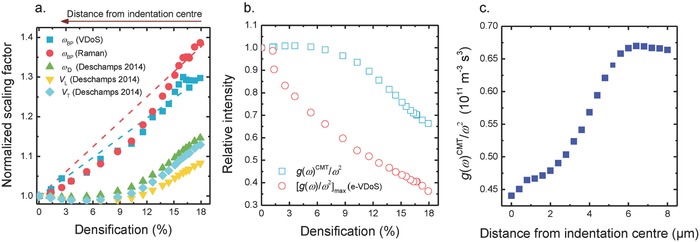
Data evaluation according to the continuous medium transformation (CMT) model. a) Variation of the scaling factors relative to Boson peak frequency ω_BP_ (VDoS), ω_BP_ (Raman), Debye frequency ω_D_, and elastic constants (*V*_T_ and *V*_L_) as a function of densification. Dashed lines in (a) are drawn to guide the eye. b) Variation of the relative intensity of *g*(ω)/ω^2^ (e‐VDoS) and *g*(ω)^CMT^/ω^2^ as functions of densification. c) Variation of *g*(ω)^CMT^/ω^2^ as a function of the distance from the center of the indenter. In (a) and (b), all data were normalized to their normal density value (corresponding to nondensified silica).

As noticed in Figure 3, indentation‐induced stress affects material density. Density variations, on the other side, affect other properties of the material, including the elastic constants.^74^ Therefore, comparison of experimental data to the predictions of theoretical models requires to consider the changes also in these parameters. Here, we now compare the Boson peak frequency  ω_BP_ and intensity (e‐VDOS) with predictions of the CMT model. First, in order to check whether the shift of the Boson Peak frequency ω_BP_ (Figure 4) can be ascribed completely to elastic medium transformations as predicted by the CMT model, we compare the dependence of Boson peak frequency ω_BP_, the Debye frequency ω_D_ 44 (from Equation (3)) and the elastic constants (*V*_T_ and *V*_L_) as functions of the density around the residual imprint. Results of this comparison are plotted in **Figure** **5**a

(3) $$\omega_{D}\;\;=\;\;\left(6\pi^{2}\rho N_{A}m/M\right){\;}^{1/3}\;V_{{\;}_{D}}$$

with the Avogadro number *N*_A_, the average number of atoms per molecule in the sample *m*, the average molar weight *M*, and the density ρ as obtained using the shift of the D2 band. *V*_D_ is the Debye sound velocity obtained by averaging *V*_L_ and *V*_T_ through ($3/V_{D}^{3})\;\;\;=\;\;\;(1/V_{L}^{3}\;\;+\;\;2/V_{T}^{3})$. Sound velocity data are taken from ref. 75.

The variation of the Boson peak intensity under mechanical stress is usually compared to the variation of the Debye level $\omega_{D}^{-3}$ (with $g_{D}(\omega )/\omega^{2}\;\;\approx \;\;\omega_{D}^{-3}$),^76^, ^77^ but computational simulation has shown the limits of this approach in the consideration of vitreous silica.^78^ In the alternative CMT model, the comparison is done between the observed *g*(ω) and a modeled *g*(ω)^CMT^.^79^ This takes into account the fact that the increase of pressure induces an increase of sound velocities and structural density, corresponding to a decrease of the VDoS^79^

(4) $$g{\left(\omega \right)}^{\mathrm{CMT}}/\;\omega^{2}=\left(1/\left(d\;\times \;V_{D}^{3}\;\right)\right)\;\;\;=\;\;{\left(3d\right)}^{-1}\;\left(\frac{1}{V_{L}^{3}}\;\;+\;\;\frac{2}{V_{T}^{3}}\right)$$

Results are plotted in Figure 5b,c.

Figure 5a reveals that the density dependence of scaling factors for the Boson peak frequency ω_BP_ (VDoS), ω_BP_ (Raman), the Debye frequency ω_D_, and the elastic constants *V*_T_ and *V*_L_ are approximately linear. Variations on the Boson peak frequency ω_BP_ (VDoS, Raman) as they are induced by indentation show a stronger density dependence than the Debye frequency or the sound velocities. A similar trend was previously seen in isostatically compressed glasses, e.g., silica,^44^, ^72^ borosilicate,^77^ germanium oxide,^80^ vitreous B_2_O_3_,^81^ or glass‐forming polymers.^76^ In Figure 5b, the dependence of the Boson peak intensity and the predicted intensity according to CMT on densification are shown. We notice that the intensity of the e‐VDOS decreases very quickly with densification, while CMT predicts a more complex trend, similar to earlier observations.^78^, ^82^ For the present case, both Figure 5a,b therefore show clearly that the Boson peak frequency shift and intensity variations induced by indentation cannot be attributed alone to modifications of a continuous elastic medium. Instead, they are related to local structural transformations^44^, ^80^ and spatial distributions of local moduli, respectively.^83^ In this context, some authors^77^, ^80^ suggested that two contributions (as described in Equation (5)) affect the position and the intensity of the Boson peak: the degree of variability of the elastic properties (caused by disorder) and to additional vibrations related to a correlation length ξ which expresses the spatial extent of variability. However, there is presently no conclusive model connecting both parameters and the density of the system

(5) $$\frac{\Delta \omega_{\mathrm{BP}}\;}{\omega_{\mathrm{BP}}\;}\;\;=\;\;\frac{\Delta V_{T}}{V_{T}}\;\;\;+\;\;\frac{\Delta \xi }{\xi }$$

The model of a noncontinuous structure (underlying Equation (5))^84^ ascribes the additional vibrations which are at the origin of the Boson peak to material heterogeneity on the nanometric scale. This draws an analogy between the low‐frequency Raman scattering spectra of glasses and heterogeneous (granular) materials containing nanoparticles.^85^ The system is assumed to consist of cohesive domains in which the atoms are strongly linked to each other, and of softer interdomain regions. The Boson peak arises from hybridization of acoustic vibrations with quasi‐localized modes around those elastic heterogeneities. The size of the elastic heterogeneities is very similar to the length‐scale of dynamic heterogeneity in the liquid state.^86^ It has been shown by molecular dynamics simulation^87^, ^88^ that the value of ξ typically corresponds to around 20–30 times of the average particle‐particle distance. For smaller ξ, classical elasticity description for deformation breakdown and a prediction of low‐frequency vibrational modes becomes very limited. For the specific case of silica glass, Leonforte et al.^89^ have shown the existence of inhomogeneous regions in Lennard‐Jones systems with ξ equal to 30 and 23 interparticle distances for 2D and 3D models, respectively.

**Figure 6:**
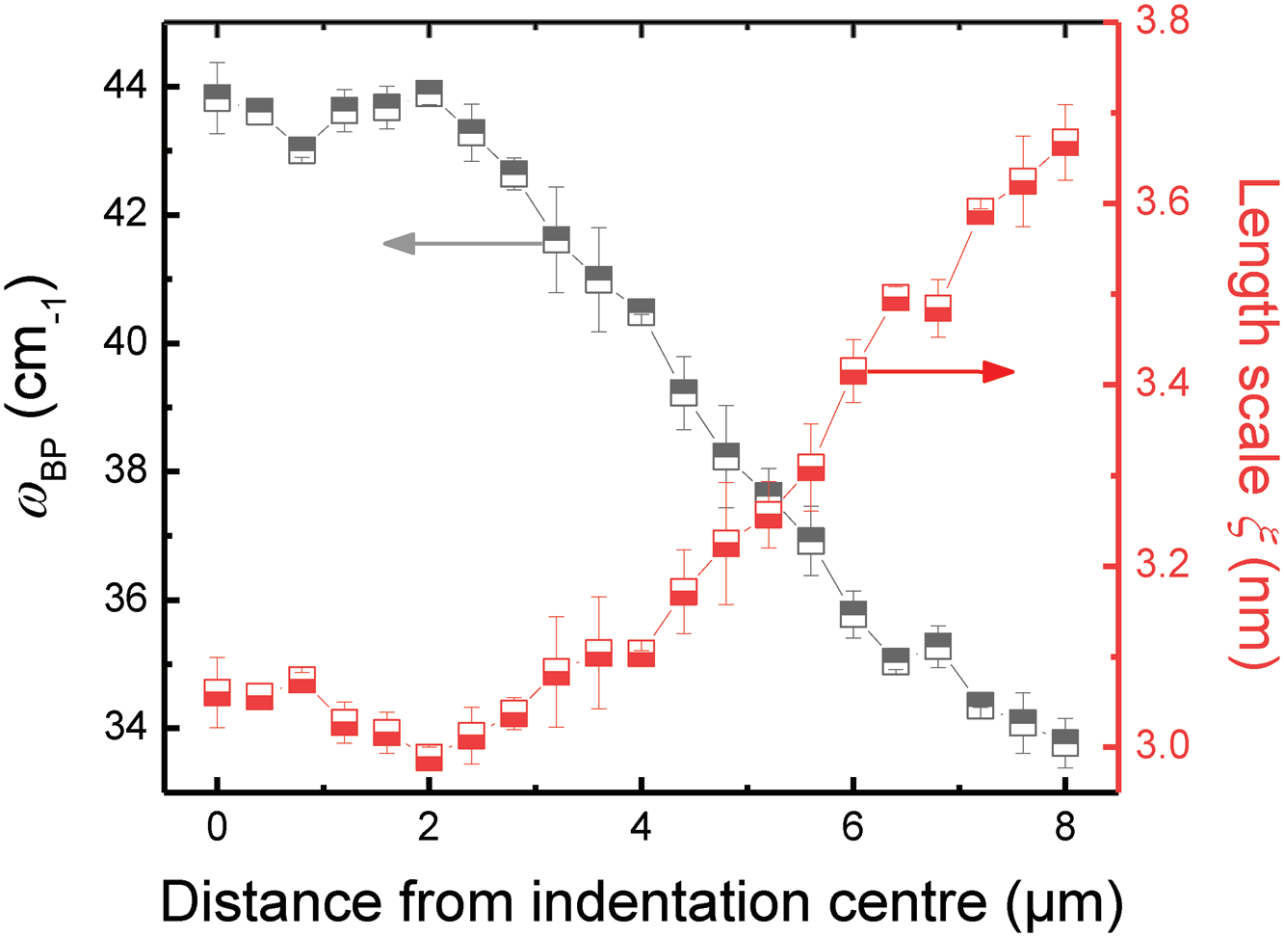
Effect of indentation‐deformation on local structural heterogeneity. Variation of Boson peak frequency ω_BP_ and the associated length scale ξ with the distance from the indentation center.

In this theoretical framework, the decrease of the VDoS in the most compacted areas during indentation deformation (Figure 4) is due to weaker hybridization. This results from a decrease of the elastic contrast between the rigid domains and the soft domains. The characteristic length scale ξ is now determined from the ratio of the transverse sound velocity *V*_T_ and ω_BP_ (**Figure** **6**). Noteworthy, this evaluation does not take into account geometric aspects except for assuming that the characteristic geometry does not vary significantly with pressure. A geometry factor *S* is sometimes employed so that ξ_corr_ =  *S* (*V*_T_/ω_BP_), with *S* = 0.8 for spherical domains or *S* = 0.5 for linear domains.^79^ Without knowing the actual shape, a mean value of *S* = 0.65 is usually used.

The value of ξ = 3.6 nm (ξ_corr_ = 2.4 nm) which we observe in the nondensified area is in good agreement with computational simulation (ξ = 3.3 nm,^89^) and experimental data (ξ = 3.74 nm^32^). We notice a significant decrease of ξ to about 3.1 nm (ξ_corr_ = 1.98 nm) toward the center of the indentation imprint. This reduction by about 18% corresponds to elastic homogenisation of the network and to the softening of the rigid domains. It also agrees with experiments of isostatic compression of glasses^33^ and polymers^90^, ^91^ which have shown that the correlation length decreases with densification in a power law dependence, $\xi \;\sim \;p^{-x}$. The value of the exponent *x* was reported to be ≤0.25, apparently dependent on liquid fragility.^90^ In Figure 6, we can distinguish two regimes: i) between 4 and 8 µm from the indentation center (at relatively low pressure, Figure 3) ξ decreases progressively, reflecting network homogenization, and ii) within the first ≈4 µm from the indentation center, ξ levels out to a constant value, mirroring the pressure loading curve shown in Figure 3. The latter behavior marks the saturation limit of the homogenization process. This is in agreement with experimental studies of isostatically compacted silica, and also with computational studies of the pressure dependence of rotational modes of connected silica tetrahedra which appear to approach a τ_+_/τ_−_ratio of unity upon pressure saturation.^78^ It is widely assumed that ξ is directly related to a structural correlation length.^92^, ^93^ A semiempirical proportionality was suggested between ξ and the inverse width of the first sharp diffraction peak (FSDP),^42^, ^92^ whereby the latter is thought to arise from the periodicity of rings of SiO_4_ tetrahedra.^94^ However, the correlation with the annular position of the FSDP and, thus, the static correlation length seems to be more complex.^95^, ^96^

## 3 Discussion

We considered the topological origin of microscopic deformation processes on glass surfaces in sharp contact situations. Through dissipation of mechanical energy, these processes (in particular, structural compaction, and shear) determine the material's resistance to flaw initiation. Acting as local stress amplifiers, such flaws are responsible for mechanical failure and, thus, the practical strength of glass products.

Initially focusing on vitreous silica as a material of technical as well as fundamental importance enabled us to exclude contributions of chemical fluctuations in data analysis. Spatially resolved Raman spectroscopy was employed in the THz‐gap in order to monitor structural variations induced by microindentation. This provided access to variations in the excess of the vibrational density of states of vitreous materials relative to Debye solids. Since the presence of such excess modes is linked to spatial fluctuations in the rigidity of the glass network, their analysis allows for probing the role of long‐ranging structural heterogeneity in the deformation process. This overcomes the limitations of previous observations of atomic interactions on short and intermediate length‐scale which could not explain the overall extent of deformation. Through scaling of low‐frequency Raman scattering maps, deformation‐induced shifts in the length‐scale of rigidity fluctuations and fluctuation contrast were revealed in the position and intensity, respectively, of the Boson peak. The extent of these variations corresponds to the degree of local structural densification. We conclude that inherent fluctuations in rigidity occurring on the scale of a few nanometers control the deformation reactions in vitreous silica and other glassy materials. While continuous medium and molecular field approximations fail in the prediction of the mechanical behavior of glasses, as a hypothesis, glasses respond to local mechanical contact in a way which is similar to that of granular media with pronounced intergranular cohesion. This puts new emphasis on recent concepts of nanoductility and weakest link theories in the description of oxide glasses^97^, ^98^ and may open a new route for tailoring glasses with extreme defect resistance, for example, through chemical tailoring of intergranular cohesion in glasses with very strong network crosslinking using threefold‐coordinated anion species^6^, ^39^, ^99^ or through adjusting ion mobility and ion–ion competition in network percolation channels.^100^, ^101^

## 4 Experimental Section

Commercial‐grade silica glass *v*‐SiO_2_ (Suprasil 2, Heraeus) specimens were indented with a Struers Duramin microindenter, using a Vickers tip with a load of 2.94 N and a constant loading time of 15 s. The load was chosen so as to create a large but crack‐free permanent imprint (Figure 2).^12^ Structural studies were conducted with a Renishaw Invia micro‐Raman spectrometer, equipped with a low‐frequency notch filter performing down to ≈10 cm^−1^. Samples were excited with a 514 nm Argon laser at ambient temperature, with a spatial resolution of <0.5 µm. The Raman signal was collected with a CCD camera over the frequency range of 10–1386 cm^−1^ with a resolution of 2 cm^−1^, using a 50 × objective. For each spectrum of a 2D map, an accumulation time of 280 s was employed.

FEM was performed on ABAQUS.^102^ The indentation process was modeled using a 2D axisymmetric model of a Vickers‐equivalent conical indenter with an opening angle of 70.29° similar to previous studies by Bruns et al.^16^ All material parameters were assumed to be rate‐insensitive and representative of room temperature values. The contact conditions between the rigid Vickers indenter and the sample surface were assumed to be frictionless. Furthermore, elastic isotropy was assumed for fused silica with an elastic modulus of 70 GPa and a Poisson ratio of 0.18.^103^ Anomalous material behavior was modeled using Drucker–Prager–Cap plasticity with a yield strength under pure shear of 7.5 GPa. Details on this model can be found elsewhere.^14^ Hereby, densification induced sigmoidal isotropic hardening and saturation of densification as reported by Rouxel et al.^104^ were included.

## Conflict of Interest

The authors declare no conflict of interest.
